# Supplementary material for: Dietary Inflammatory Index and the Risk of Hyperuricemia: A Cross-Sectional Study in Chinese Adult Residents
Source: Nutrients. 2021 Dec 16;13(12):4504. doi: 10.3390/nu13124504 (PMC8708184; doi:10.3390/nu13124504)
Supplement: Supplementary file 1 [file nutrients-13-04504-s001.zip › nutrients-1476706-supplementary.pdf]

Table S1. Characteristics of subjects according to quartiles of the DII score.

Characteristics	Quartiles of the DII			
	Q1 (n = 1996)	Q2 (n = 1972)	Q3 (n = 1928)	Q4 (n = 1983)
Min and Max of DII	-0.49 to 2.3	-1.3 to -0.49	-1.3 to-2.1	-2.1 to -4.0
Age (years)	53.0 (16.3)	50.9 (14.9)	50.0 (14.4)	48.9 (13.6)
Hyperuricemia				
Non-hyperuricemia	1612(80.8%)	1657(84.0%)	1639(85.0%)	1737(87.5%)
Hyperuricemia	384(19.2%)	316(16.0%)	289(15.0%)	246(12.5%)
Gender				
Male	735(37%)	886(45%)	926(48%)	1081(55%)
Female	1261(63%)	1087(55%)	1002(52%)	902(45%)
Marital status				
Single	106(5%)	102(5%)	105(5%)	113(6%)
Married	1603(80%)	1670(85%)	1651(86%)	1756(89%)
Other	287(14%)	201(10%)	172(9%)	114(6%)
Region				
Urban	785(39%)	745(38%)	787(41%)	896(45%)
Rural	1211(61%)	1228(62%)	1141(59%)	1087(55%)
Education				
None	607(30%)	519(26%)	397(21%)	334(17%)
Grad from primary	382(19%)	397(20%)	408(21%)	381(19%)
Lower middle school degree	602(30%)	634(32%)	663(34%)	691(35%)
Upper middle school degree and above	215(11%)	212(11%)	214(11%)	273(14%)
Smoke				
Nonsmoker	1482(74%)	1356(69%)	1313(68%)	1303(66%)
Smoker	514(26%)	617(31%)	615(32%)	680(34%)
BMI (kg/m^2)				
≤18.5	146(7%)	130(7%)	112(6%)	89(4%)
18.5-24	1093(55%)	1044(53%)	1024(53%)	1058(53%)
24-28	565(28%)	601(30%)	613(32%)	632(32%)
≥28	192(10%)	198(10%)	179(9%)	204(10%)

DII: dietary inflammatory index; BMI: body mass index.

Table S2. Odds ratios (OR) and 95% confidence intervals for hyperuricemia by DII scores.

	**Model 1**	**P**	**Model 2**	**P**
Age, per 10 years	1.19(1.14-1.25)	<0.01	1.18(1.11-1.25)	<0.01
Gender				
Male	1 (ref)		1 (ref)	
Female	0.51(0.45-0.58)	<0.01	0.53(0.45-0.63)	<0.01
DII				
Q1	1 (ref)		1 (ref)	
Q2	0.80(0.68-0.95)	0.01	0.83(0.70-0.99)	0.03
Q3	0.69(0.58-0.82)	<0.01	0.72(0.60-0.86)	<0.01
Q4	0.69(0.58-0.82)	<0.01	0.73(0.61-0.88)	<0.01
Marital status				
Single			1 (ref)	
Married			0.69(0.51-0.94)	0.02
Other			0.91(0.64-1.32)	0.63
Region				
Urban			1 (ref)	
Rural			0.76(0.66-0.87)	<0.01
Education				
None			1 (ref)	
Grad from primary			0.88(0.72-1.07)	0.21
Lower middle school degree			0.88(0.72-1.07)	0.20
Upper middle school degree and above			0.95(0.74-1.21)	0.67
Current smoke				
No			1 (ref)	
Yes			0.98(0.83-1.15)	0.68
BMI (kg/m2)				
18.5-24			1 (ref)	
≤18.5			0.56(0.38-0.8)	<0.01
24-28			1.91(1.66-2.19)	<0.01
≥28			3.11(2.57-3.75)	<0.01

DII: dietary inflammatory index; BMI: body mass index.

Table S3. Odds ratios (OR) and 95% confidence intervals stratified by gender.

	**Male**		**Female**	
	**OR (95%CI)**	**P**	**OR (95%CI)**	**P**
Age, per 10 years	0.97 (0.9-1.04)	0.40	1.62 (1.47-1.78)	<0.01
DII				
Q1	1 (ref)		1 (ref)	
Q2	1.00 (0.81-1.24)	1.00	0.63 (0.47-0.84)	<0.01
Q3	0.77 (0.61-0.97)	0.03	0.64 (0.48-0.84)	<0.01
Q4	0.75 (0.58-0.95)	0.02	0.66 (0.50-0.87)	<0.01
Marital status				
Single			1 (ref)	
Married	0.88 (0.63-1.25)	0.48	0.52 (0.28-1.06)	0.05
Other	1.08 (0.67-1.74)	0.76	0.54 (0.27-1.17)	0.10
Region				
Urban			1 (ref)	
Rural	0.75 (0.62-0.9)	<0.01	0.79 (0.63-0.99)	0.04
Education				
None			1 (ref)	
Grad from primary	0.92 (0.68-1.24)	0.57	0.99 (0.74-1.32)	0.95
Lower middle school degree	0.78 (0.59-1.05)	0.09	1.27 (0.94-1.71)	0.11
Upper middle school degree and above	0.98 (0.73-1.34)	0.91	1.16 (0.82-1.64)	0.39
Current smoke				
No		0.47	1 (ref)	
Yes	0.94 (0.79-1.12)		0.82 (0.5-1.28)	0.39
BMI (kg/m2)				
18.5-24			1 (ref)	
≤18.5	0.52 (0.30-0.83)	0.01	0.67 (0.38-1.12)	<0.14
24-28	2.03 (1.69-2.43)	<0.01	1.63 (1.30-2.05)	<0.01
≥28	2.61 (1.99-3.42)	<0.01	3.37 (2.56-4.41)	<0.01

DII: dietary inflammatory index; BMI: body mass index.
